# High-Efficiency PDLC Smart Films Enabled by Crosslinking Agent Optimization and MoS_2_ Nanosheets for Energy-Saving Windows

**DOI:** 10.3390/ma18225139

**Published:** 2025-11-12

**Authors:** Tao Yu, Fuman Jing, Yingjie Shi, Zhou Yang, Jianjun Xu, Zuowei Zhang, Meina Yu, Huai Yang

**Affiliations:** 1 School of Materials Science and Engineering, University of Science and Technology Beijing, Beijing 100083, China; 2 Institute for Advanced Materials and Technology, University of Science and Technology Beijing, Beijing 100083, China; 3 School of Materials Science and Engineering, Peking University, Beijing 100871, China

**Keywords:** polymer dispersed liquid crystal, smart window, MoS_2_, radiative cooling

## Abstract

**Highlights:**

**What are the main findings?**

- The structure of the PDLC network is greatly influenced by the alkyl chains of the polymer monomers.
- By introducing MoS_2_ nanosheets, the PDLC film has a low drive voltage (23 V) and high CR (135).
- The MoS_2_/PDLC films can reduce the temperature by about 5 °C compared with the conventional films

**What are the implications of the main findings?**

- The E-O properties of PDLC film can be improved by adjusting the structure of the polymer molecules.
- Appropriate amounts of nanoparticles can optimize the E-O properties of PDLC film.
- MoS_2_ can enhance the heat insulation performance of PDLC film.

**Abstract:**

Polymer-dispersed liquid crystal (PDLC), as an electrically controlled dimming material, has broad application prospects in various fields, including smart glass, display technology, and optical devices. However, traditional PDLC materials still face some challenges in practical applications, such as a high driving voltage and insufficient optical contrast, which limit their further application in high-performance optoelectronic devices. In this study, PDLC composite films exhibiting low-voltage operation (23 V), high contrast ratios (135), and rapid response times (T_R_ ~1.28 ms, T_D_ ~48 ms) were developed. This was achieved by modifying the chain length of the crosslinking agent and polymer monomer as well as by incorporating molybdenum disulfide (MoS_2_) nanosheets. It shows a good regulation ability in the sunlight range (ΔT_sol_ = 63.92%, ΔT_lum_ = 73.97%). Simultaneously, the various chemical bonds inside the film and its special network structure enable it to exhibit a good radiative cooling effect. The indoor sunlight simulation tests showed that the indoor temperature decreased by 5 °C. This study provides valuable ideas for the development and preparation of smart windows with high efficiency and energy savings.

## 1. Introduction

Building energy consumption is the main source of global energy consumption and carbon emissions. According to reports, global building energy consumption accounts for approximately 30% of total energy [1,2,3]. Data from China show that the proportion of building energy consumption can reach approximately 20–50%. As the process of urban modernization accelerates, this proportion continues to rise rapidly. Among these, doors and windows are the main components of building energy consumption [4,5]. In China, the building energy consumption of doors and windows accounts for more than 40–50% of the total energy consumption (especially in the northern heating areas), which is a weak link to building energy conservation. Doors and windows are key leverage points for building energy conservation. Both the world and China are promoting energy efficiency through technological upgrades and policies. Smart windows are a type of current window technology that can dynamically adjust the transmittance, thermal insulation performance, etc. [6,7,8,9], based on the external environment. At present, mainstream technologies are divided into electrochromic, thermochromic, photochromic, suspended particle, and Polymer-dispersed liquid crystal (PDLC) smart windows. Among them, PDLC has developed rapidly owing to its advantages, such as rapid response, environmental protection, pollution-free, and large-scale preparation [10,11,12,13,14,15,16,17].

As an electrically controlled dimming material, PDLC has shown broad application prospects in smart glass, display technology, optical devices, etc. [18,19,20]. PDLC material is composed of liquid crystal droplets dispersed in a polymer matrix. Its core characteristic is to control the orientation of liquid crystal molecules through an external electric field, thereby achieving rapid switching of the material between transparent and opaque states [21,22]. Under external electric field, the liquid crystal molecules tend to align to the field direction, and the refractive index of the liquid crystal droplets matches that of the polymer. Consequently, light is transmitted. When the electric field is removed, the liquid crystal droplets become randomly arranged, causing a refractive index mismatch and thereby a light-scattering state. However, traditional PDLC materials still face challenges in practical applications, such as high driving voltage, slow response speed, and insufficient optical contrast, which limit their further application in high-performance optoelectronic devices. Researchers have adjusted the electro-optical properties of PDLC films by adjusting polymer monomers, liquid crystal monomers, etc. [23,24,25,26,27]. Zhang et al. [28] prepared PDLC films containing monomers with different alkyl chain lengths via nucleophilic thiol binding reactions. The effects of the monomer alkyl chain length, dye, and temperature on the electro-optical properties of the PDLC films were investigated. The study found that the alkyl chain length and polymerization rate of the monomer jointly determine the size of the liquid crystal droplets, thereby affecting the electro-optical properties of the liquid crystal. Sun et al. [29] used UV-induced polymerization to prepare PDLC films with acrylate and thiol monomers. Adding an appropriate concentration of the tetrafunctional thiol monomer to the PDLC system resulted in a lower driving voltage and higher CR. Lin et al. [30]. systematically studied the effects of methacrylate monomers with different end groups (methyl, hydroxyl, epoxy, tetrahydrofuran, cyclohexane, benzene and siloxane) on the morphology, optoelectronic properties and mechanical properties of PDLC composite films, they found that the addition of hydroxyl groups significantly improved the mechanical properties of the film.

In recent years, the rise of nanotechnology has provided new ideas for optimizing the performance of PDLC materials [31,32,33]. By introducing nanoparticles into PDLC systems, researchers have found that their electro-optical properties, mechanical properties, and stability can be significantly improved. Nanoparticle doping not only reduces the driving voltage of the PDLC but also improves its response speed and optical contrast. For example, the introduction of nanoparticles such as titanium dioxide (TiO_2_) and silicon dioxide (SiO_2_) can enhance the dielectric anisotropy of PDLC, thereby optimizing their electro-optical response characteristics. In addition, the surface modification and functionalization of nanoparticles further expand the multifunctionality of PDLC, such as their photocatalytic and self-cleaning properties [34,35,36,37]. The molybdenum sulfide nanosheets (MoS_2_) have a two-dimensional layered structure with a large specific surface area, which can form a large number of interfaces when combined with other materials. These interfaces can cause multiple reflections, scattering and refraction of the incident light during its propagation inside the material, which can increase the propagation path of the light inside the film. Therefore, in this research, MoS_2_ were doped into PDLC films, aiming to increase the scattering center within the film and reduce its light transmittance in the off state, thereby improving its CR. The nanosheet also has excellent mechanical strength, which can maintain its stability in PDLC films.

Radiative cooling is a passive cooling technology that does not require external energy input and directly dissipates heat into outer space through infrared radiation in a specific band. In recent years, a large number of studies have been reported on the research of smart windows in radiative cooling, which has significantly cut down the energy consumption of windows. Zhang et al. prepared a film with high radiative cooling efficiency by introducing fluorinated monomers into the PDLC film, which reduced the indoor temperature by 3.5 °C compared with the original film [38]. In addition, Zhang et al. adjusted liquid crystal monomers to prepare a film with an emissivity of approximately 95% in the atmospheric window, showing an extremely high radiative cooling efficiency [39]. Deng et al. prepared a reversible smart window by introducing SiO_2_ into a PDLC film, and the film emissivity was dynamically controlled by dynamic reversal [40]. The rich chemical bonds inside the PDLC film, such as C-H, C-O, and C=O, and its special micro-network structure, have a higher emissivity at 8–13 μm, giving the film a higher radiative cooling efficiency, which greatly improves the film’s energy-saving performance [39,40,41,42].

In this study, by adjusting the chain length of the crosslinker, the chain length of the polymerized monomer, and introducing MoS_2_ nanosheets into the PDLC system, a PDLC film with low voltage and high CR was prepared. The driving voltage was 23 V and the CR reached 135. The sunlight simulation test shows that it can reduce the indoor temperature by 5 °C compared with normal glass. This research opens up broad possibilities for the development of smart windows with superior solar heating regulation capabilities and excellent radiative cooling functions.

## 2. Materials and Methods

### 2.1. Synthesis of MoS_2_ Nanosheets

The MoS_2_ nanosheets were synthesized using a hydrothermal method. First, 450 mg of ammonium molybdate was dissolved in 35 mL of distilled water and then 12 g of thiourea was added to dissolve the solution. Oxalic acid (500 mg) was added to the clear solution and stirred continuously to adjust the pH to approximately 7.5. The solution was then transferred to a 100 mL reactor and placed in an incubator, and the temperature was adjusted to 200 °C for 24 h. After the reaction was complete, it was washed three times with water and ethanol. The resulting black precipitate was dried in a vacuum oven at 75 °C for 12 h to obtain MoS_2_ nanosheets.

### 2.2. Preparation of PDLC Film

**Figure 1:**
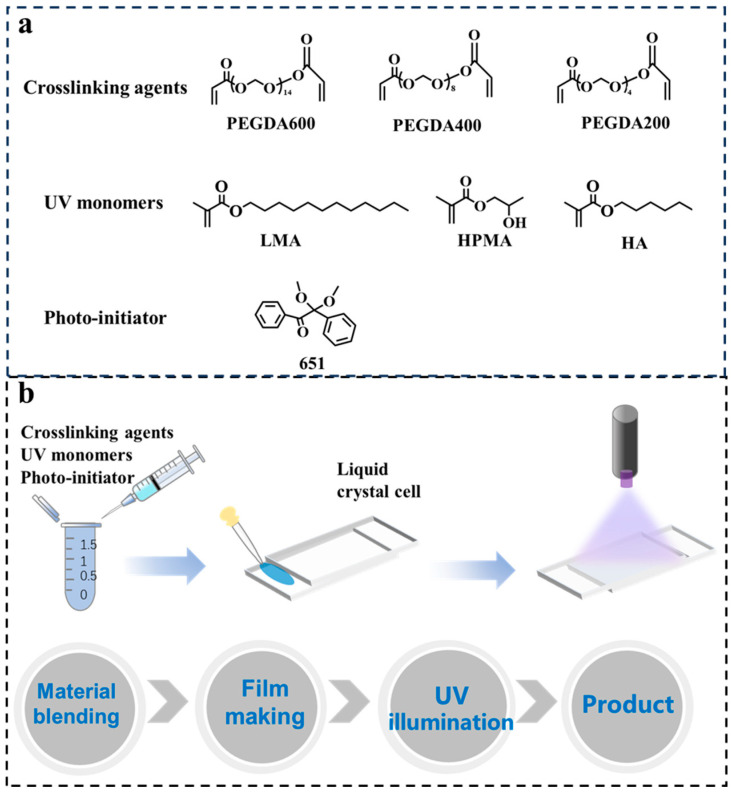
(**a**) Chemical structures of material. (**b**) Schematic diagram of the preparation process of PDLC film.

Figure 1b shows the preparation scheme of the PDLC film. Firstly, the polymerization monomer, crosslinking agent, LC, and photo-initiator were mixed in the proportions specified in Table 1. The mixture needs to be stirred thoroughly on an oscillator until it becomes a clear, uniform solution. Next, it was introduced in the LC cell (gap: 20 μm) by capillarity, and then exposed to external light (intensity = 15 W/cm^2^, wavelength = 365 nm) for 2 min. The preparation of the other samples follows the same procedure as described above. All chemical reagents have not been purified further, and the chemical structure is shown in Figure 1a. Details of all experimental materials and the research methods are provided in the Supplementary Material.

## 3. Results and Discussion

### 3.1. Effects of Crosslinker Chain Length and Monomer Chain Length on the Electro-Optical Properties of Thin Films

**Figure 2:**
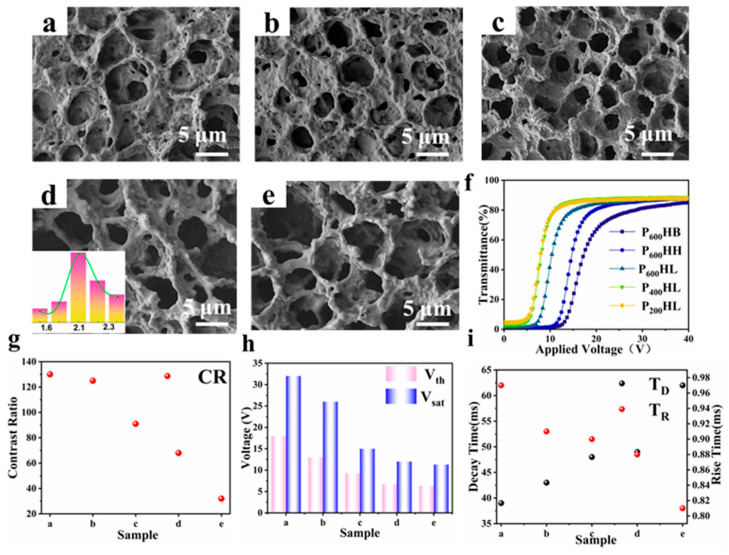
Micrographs of the polymer networks of samples (**a**) a, (**b**) b, (**c**) c, (**d**) d, (the insert is the pore diameter of the film) and (**e**) e; (**f**) the voltage-dependent transmittance curves; (**g**) CR of a–e samples; (**h**) Vth and V_sat_ of a–e samples; (**i**) T_R_ and T_D_ of samples a–e.

This group of experiments discussed the effects of crosslinkers and monomers with different chain lengths on the film microstructure. Among them, P600HB, P600HH, P600HL, P400HL, and P200HL correspond to samples a, b, c, d, and e, respectively. The microstructures and the electro-optical curves for the five sample groups are shown in Figure 2a–e.

From samples a, b, and c, it can be seen that when the crosslinker chain length is constant, as the polymerized monomer chain length increases, the mesh of the film gradually becomes sparse, and the size tends to increase. Compared with samples a and b, the mesh structure of sample C is more uniform. This is because increasing the chain length leads to an increase in molecular weight, thereby reducing the number of reactive molecules and affecting crosslinking kinetics. In addition, the steric hindrance of BA monomers with shorter chain lengths is smaller. As the chain length shortens, more reactive groups exist in the system because at the same mass fraction, the molar fraction of short-chain monomers is larger, which accelerates the reaction rate of the system, shortens the diffusion and aggregation time of LC molecules, and reduces the size of the polymer mesh. Comparing samples c, d, and e, it was found that when the polymer monomers were the same, the mesh size tended to decrease with an increase in the crosslinking agent chain length. The increase in the chain length of the crosslinker will cause the molecular chain to curl and coil, which hinders the diffusion and aggregation of the liquid crystal molecules, forming a small polymer mesh. The internal illustration of Figure 2d shows the mesh size corresponding to sample d, The mesh size of the samples was moderate (1.6–2.3 µm).

Figure 2f shows the curves of transmittance variation with voltage for the five samples. Comparing the samples a, b, and c, the driving voltage of sample c is the lowest. This is because the increase in mesh holes reduces the anchoring force of the mesh holes on the liquid crystal droplets, thereby reducing the driving voltage, which is consistent with the SEM image. Comparing the samples in groups c, d, and e, we can see that as the chain length of the cross-linker increases, the driving voltage also increases. Figure 2g shows the CR of the sample, which was mainly related to the mesh size of the sample. The smaller the mesh, the higher the CR. The smaller the mesh, the higher the degree of scattering of light in the off state, increasing the CR of the film. The Vth and V_sat_ values of the samples are shown in Figure 2h. The response time changes in the samples are shown in Figure 2i. In a comprehensive comparison, sample d exhibited the best electro-optical performance. Its driving voltage is low; however, its CR is poor. For this reason, we introduced nanoparticles to further improve the electro-optical performance and discussed the effects of different concentrations of nanoparticles on the film micromorphology and electro-optical performance.

### 3.2. Characterization of MoS_2_ Nanosheets

**Figure 3:**
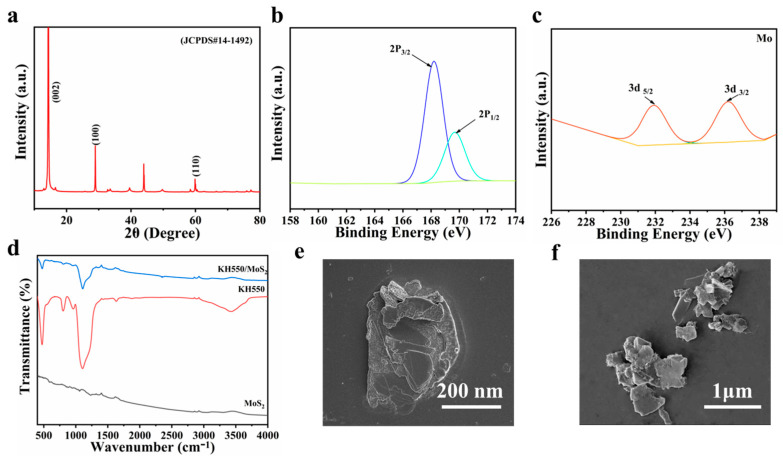
(**a**) XRD patterns; (**b**) S 2p spectrum; (**c**) Mo 3d spectrum; (**d**) FT-IR spectra of KH550, MoS_2_ and KH550/MoS_2_; (**e**,**f**) SEM micrographs of MoS_2_.

MoS_2_ nanosheets were successfully synthesized by a hydrothermal method. Figure 3a shows the XRD patterns of the MoS_2_ nanosheets. The peaks at 14.13, 29.87, and 59.25° correspond to the (002), (100), and (110) crystal planes, respectively. This is consistent with PDF#14-1492, indicating the successful synthesis of the MoS_2_ nanosheets. The chemical elements of the nano-surface were analyzed by XPS, as shown in Figure 3b,c. The S spectrum of MoS_2_ is shown in Figure 3b. Two peaks are located at 168 eV and 169.5 eV, corresponding to the 2p_3/2_ and 2p_1/2_ respectively. The peaks at 231.4 eV for Mo-3d_5/2_ and 236 eV for Mo-3d_3/2_ indicate that Mo exhibits a Mo^3+^ state in MoS_2_. To improve the dispersibility of MoS_2_ nanosheets in PDLC films and disperse the nanoparticles in the polymer phase as much as possible, the surface of MoS_2_ was modified with γ-aminopropyl triethoxysilane (KH550), and its surface functional groups were analyzed by infrared spectroscopy. Figure 3d presents the Fourier transform infrared spectra of the original MoS_2_, KH550/MoS_2_ composite material, and KH550. By comparing the spectral curves of the three samples, it is evident that the characteristic absorption peak signal of the original MoS_2_ is weak and lacks a distinct characteristic peak shape. In the spectrum of the KH550/MoS_2_ composite material, two new characteristic absorption peaks have been added compared to pure MoS_2_, located at 1105 cm^−1^ and 489 cm^−1^ wavenumbers, respectively, which corresponds to the characteristic peaks from KH550. This result confirms that an effective interaction has been formed between KH550 and MoS_2_, and a successful recombination has been achieved. In addition, Figure 3f shows the overall microscopic morphology of the original MoS_2_ sample, and Figure 3e is a high-magnification image of the corresponding area. From the magnified images, it can be clearly observed that MoS_2_ presents a typical two-dimensional lamellar structure, with complete lamellar morphology, and the cross-sectional average sizes range from 100 to 200 nm.

### 3.3. Effects of MoS_2_ Nanosheets on Film Morphology and Electro-Optical Properties

**Figure 4:**
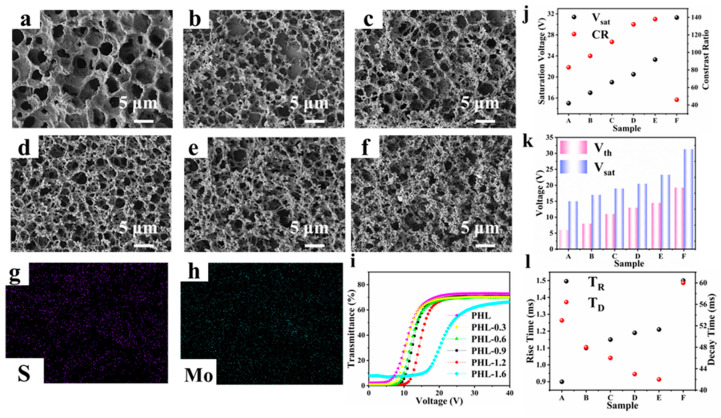
Micrographs of the polymer networks of samples (**a**) A, (**b**) B, (**c**) C, (**d**) D, (**e**) E and (**f**) F; (**g**,**h**) the EDS mapping images; (**i**) the voltage-dependent transmittance curves; (**j**) V_sat_ and CR of A–F samples; (**k**) Vth and V_sat_ of A–F samples; (**l**) T_R_ and T_D_ of samples A–F.

The changes in the microstructure and the effect on the electro-optical properties of the films after adding different proportions of MoS_2_ nanosheets were studied. Figure 4a–f shows schematic diagrams of the scanned images of the samples. The samples were PHL, PHL-0.3, PHL-0.6, PHL-0.9, PHL-1.2, and PHL-1.6, which were named A, B, C, D, E, and F, respectively. Their compositions are listed in Table 1. The meshes of all samples doped with MoS_2_ nanosheets showed a decreasing trend compared with the original sample, and the mesh surface showed many dense protrusions. With an increase in the MoS_2_ proportion, the mesh changed from relatively small and uniform to small and agglomerated. This is because as the mass fraction of nanosheets increases, the van der Waals interaction between nanoparticles is enhanced, resulting in agglomeration, as shown in Figure 4e. To show that the MoS_2_ nanosheets were successfully attached to the surface of the polymer network, EDS elemental analysis was performed, as shown in Figure 4g,h. Both S and Mo are detected in the polymer network. The results show that the MoS_2_ nanosheets were successfully embedded in the PDLC film. The ratio distribution and scanning position of each element are shown in Figure S2 and Table S1.

The electro-optical performance of the samples is shown in Figure 4i,l. Figure 4i presents the transmittance-voltage curves of the samples. It can be observed that the driving voltage of all samples increases with the addition of nanosheets. This is because the mesh size decreases, leading to stronger anchoring forces on the liquid crystal droplets. In contrast, the CR of the samples exhibits an increasing trend. The reduced mesh size enhances sunlight scattering by the film sample in the off-state, resulting in lower off-state transmittance and thus a higher CR, as shown in Figure 4j.

It is worth noting that when its mass fraction is too high, the mesh size is too densely concentrated, so that while the voltage increases, its transmittance in the on state will also decrease, which will cause a serious reduction in the CR. Figure 4k shows the Vth and V_sat_ of the sample. The corresponding time variation of the sample is shown in Figure 4l. As the mesh size decreases, its anchoring force on the liquid crystal droplet increases, which will increase T_R_, and reduce T_D_. Therefore, sample D (MoS_2_ 0.9 wt%) shows good electro-optical performance, with a driving voltage of 23 V and a CR of about 135.

### 3.4. Analysis of Optical and Thermal Management Properties of Films

**Figure 5:**
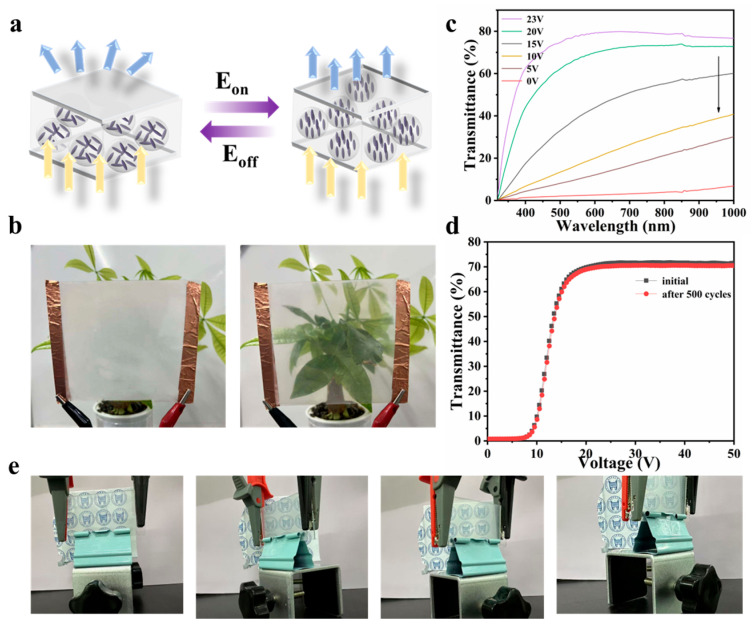
(**a**) The schematic diagram of PDLC smart window; (**b**) photos of PDLC-based smart window; (**c**) UV-visible transmittance spectra of PDLC smart window were measured at different voltages; (**d**) the transmittance of the spectra of the initial film and the film after 500 cycles; (**e**) photo display of PDLC film from different viewing angles.

The PDLC film consists primarily of a polymer matrix and liquid crystal droplets. In the off-state, the random orientation of liquid crystal molecules within the polymer matrix creates refractive index mismatching, resulting in light scattering that gives the film a milky white appearance. Upon application of an external electric field, the liquid crystal molecules align uniformly, causing the refractive index of the droplets to match that of the polymer matrix, thereby rendering the film transparent (Sample D). Figure 5a schematically illustrates this switching mechanism. Corresponding optical photographs of the film in transparent and off-states are presented in Figure 5b. Notably, the film exhibits voltage-dependent sunlight modulation capability (Figure 5c): at 0 V, the film remains in the off-state, while increasing voltage progressively enhances its transmittance across the 350–2500 nm spectral range. The modulation performance of a 20 μm-thick film was quantitatively evaluated under various voltages (Figure 5c), demonstrating multi-level daylight regulation capacity. Key parameters calculated using Equations (S1)–(S4) (Supporting Information) are summarized in Table 2. The film shows ultralow transmittance at 0 V, with T_lum_ and T_sol_ values of 1.86% and 3.86%, respectively. When driven at 23 V, these values increase significantly to 75.83% (T_lum_) and 67.78% (T_sol_). In order to verify the stability of the film, we conducted a switching state cycle test on it. Figure 5d shows the original electro-optical curve of the film and the electro-optical curve after 500 cycles. It can be found that it has hardly changed, indicating that it has good stability. In addition, the viewing angle of the film was tested. As shown in Figure 5e, it shows an excellent transmittance effect regardless of direct viewing or side viewing. The changes in transmittance at different viewing angles are shown in Figure S3. The transmittance remains at 60% at 60°.

**Figure 6:**
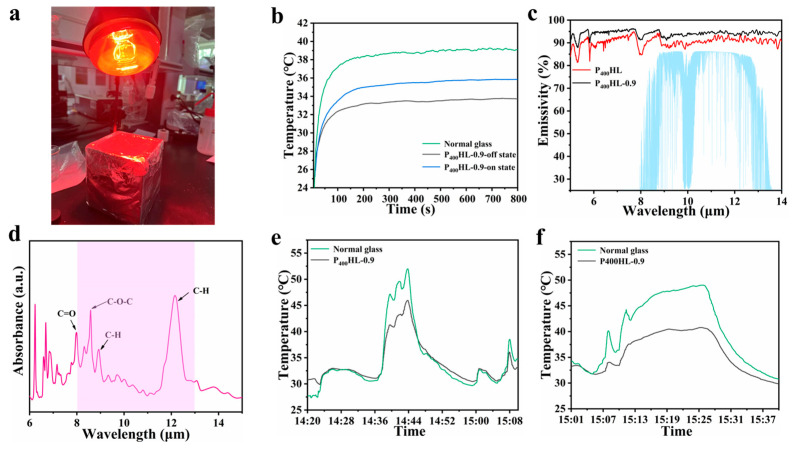
(**a**) Simulated sunlight irradiation test temperature device; (**b**) temperature change curve; (**c**) emissivity curve; (**d**) absorbance spectrum of PDLC film with ATR-FTIR spectroscopy; (**e**,**f**) outdoor test temperature change curve.

Finally, the thermal insulation and radiation cooling properties of the films are analyzed. a 300 W NIR lamp illuminated on the PDLC film (sample D) and the plain glass surface, and the temperature inside the box was tested and recorded, as shown in Figure 6a,b. The temperature inside the box covering the PDLC film was 3 °C lower than that of ordinary glass for the same irradiation time and under the condition of electricity (23 V). In addition, the temperature inside the box covered with the PDLC film is even lower, about 5 °C, when the film is not energized, mainly due to the scattering state of the film and the greatly reduced hot light entering the silo through the film. The emissivity of this PDLC film in the atmospheric window is tested and analyzed in the following, as shown in Figure 6c. The emissivity of both films in the 8–13 μm band is greater than 90%, which is due to the abundant chemical bonds inside the polymer and its porous structure (as shown in Figure 6d). The higher emissivity of the MoS_2_-doped PDLC film (P400HL0.9) compared to the undoped MoS_2_ PDLC film (P400HL) may be due to the increased scattering center in the system caused by the addition of nanoparticles. To verify its radiative cooling effect, we tested raw glass and MoS_2_-doped PDLC films (P400HL0.9) outdoors, as shown in Figure 6e and f (The peak in the figure is caused by the instantaneous change in sunlight intensity). Compared with the ordinary glass, the P400HL0.9 film shows a good radiative cooling effect, and its internal temperature is reduced by about 5 °C compared with the temperature in the chamber of the original glass.

## 4. Conclusions

In summary, by adjusting the chain length of the cross-linking agent and the polymerized monomer, and introducing MoS_2_ nanosheets, a low-voltage, high-contrast, and good thermal-regulating PDLC film was developed and prepared. When the MoS_2_ doping concentration in the system is 0.9 wt%, the film exhibits excellent electro-optical properties, with a V_sat_ of ~23 V, CR = 135 (an improvement of 38%), T_R_ = 1.62 ms, and T_D_ = 33.5 ms (an improvement of 30%). In addition, the film has good solar spectrum modulation capabilities (ΔT_sol_ = 63.92%, ΔT_lum_ = 73.97%). At the same time, the dynamic regulation of sunlight can be achieved by adjusting the voltage. In the indoor daylight simulation test, the PDLC film can reduce the temperature by about 5 °C compared with the conventional film. In addition, the rich chemical bonds and its micro-network structure enable it to show an extremely high emissivity of ~95% at 8–13 μm and have a good radiative cooling effect. This study provides a valuable method for the development of multifunctional composite films in the field of smart windows.

## Figures and Tables

**Table 1:** The compositions of the samples.

Sample	Composition	(wt. %)
Group 1: Effects of crosslinker chain length and monomer chain length on the electro-optical properties of films		
a	PEGDA600/HPMA/BA/LC	6.0/6.0/18.0/70
b	PEGDA600/HPMA/HA/LC	6.0/6.0/18.0/70
c	PEGDA600/HPMA/LMA/LC	6.0/6.0/18.0/70
d	PEGDA400/HPMA/LMA/LC	6.0/6.0/18.0/70
e	PEGDA200/HPMA/LMA/LC	6.0/6.0/18.0/70
Group 2: Effects of MoS_2_ nanosheets on film morphology and electro-optical properties		
A	PEGDA400/HPMA/LMA/MoS_2_/LC	6.0/6.0/18.0/0/70
B	PEGDA400/HPMA/LMA/MoS_2_/LC	6.0/6.0/17.7/0.3/70
C	PEGDA400/HPMA/LMA/MoS_2_/LC	6.0/10.0/17.4/0.6/70
D	PEGDA400/HPMA/LMA/MoS_2_/LC	6.0/10.0/17.1/0.9/70
E	PEGDA400/HPMA/LMA/MoS_2_/LC	6.0/10.0/16.8/1.2/70
F	PEGDA400/HPMA/LMA/MoS_2_/LC	6.0/10.0/16.4/1.6/70

**Table 2:** Optical properties of PDLC film (20 μm) under different voltages.

Transmittance	0 V	5 V	10 V	15 V	20 V	23 V
Tlum (%)	1.86	2.66	14.54	36.67	54.43	75.83
Tsol (%)	3.86	19.43	39.31	55.36	58.46	67.78
ΔTlum (%)	-	0.80	12.68	34.81	52.57	73.97
ΔTsol (%)	-	15.57	35.45	51.50	54.60	63.92

## Data Availability

The original contributions presented in this study are included in the article/supplementary materials. Further inquiries can be directed to the corresponding authors.

## Supplementary Materials

The following supporting information can be downloaded at: https://www.mdpi.com/article/10.3390/ma18225139/s1.
