# Correction: Yu et al. High-Efficiency PDLC Smart Films Enabled by Crosslinking Agent Optimization and MoS_2_ Nanosheets for Energy-Saving Windows. *Materials* 2025, *18*, 5139

**DOI:** 10.3390/ma19132841

**Published:** 2026-07-03

**Authors:** Tao Yu, Fuman Jing, Yingjie Shi, Zhou Yang, Jianjun Xu, Zuowei Zhang, Meina Yu, Huai Yang

**Affiliations:** 1 School of Materials Science and Engineering, University of Science and Technology Beijing, Beijing 100083, China; yutaouk@sina.com (T.Y.); yangz@ustb.edu.cn (Z.Y.); jjxu1109@163.com (J.X.); 2 Institute for Advanced Materials and Technology, University of Science and Technology Beijing, Beijing 100083, China; m202311376@xs.ustb.edu.cn (F.J.); syj0625@163.com (Y.S.); 3 School of Materials Science and Engineering, Peking University, Beijing 100871, China

In the original publication [1], there was a mistake in Figure 2 as published. The corrected Figure 2 is shown below:

Correspondingly, the citation of Figure 2 in Section 3.1. paragraph 2 has been corrected. The sentence “The internal illustration of Figure 2d shows the mesh size corresponding to sample d, The mesh size of the samples was moderate (1.6–2.3 µm).” should be updated to “The internal illustration of Figure 2d shows the mesh size corresponding to sample d, The mesh size of the samples was moderate (1.5–4.5 µm).”

The authors state that the scientific conclusions are unaffected. This correction was approved by the Academic Editor. The original publication has also been updated.

## Figures and Tables

**Figure 2:**
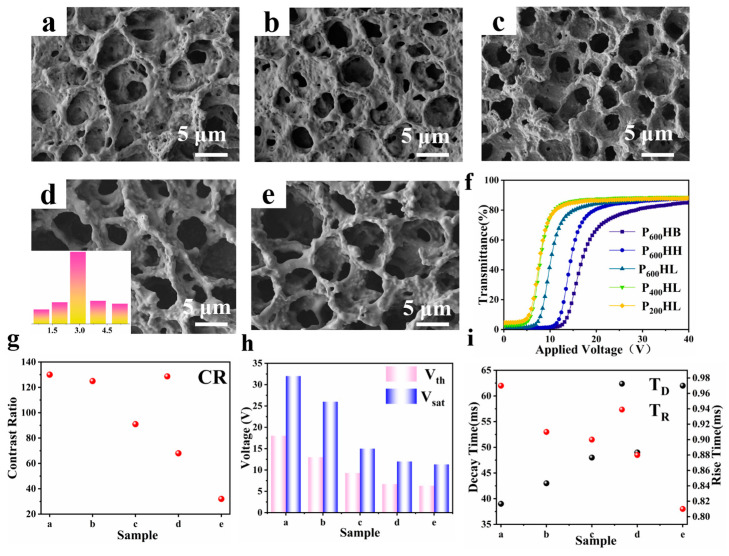
Micrographs of the polymer networks of samples (**a**) a, (**b**) b, (**c**) c, (**d**) d (the insert is the pore diameter of the film), and (**e**) e; (**f**) the voltage-dependent transmittance curves; (**g**) CR of a–e samples; (**h**) Vth and V_sat_ of a–e samples; (**i**) T_R_ and T_D_ of samples a–e.
