# Supplementary material for: High-Efficiency PDLC Smart Films Enabled by Crosslinking Agent Optimization and MoS2 Nanosheets for Energy-Saving Windows
Source: Materials (Basel). 2025 Nov 12;18(22):5139. doi: 10.3390/ma18225139 (PMC12654132; doi:10.3390/ma18225139)
Supplement: Supplementary file 1 [file materials-18-05139-s001.zip › materials-3951616-supplementary.pdf]

High-Efficiency PDLC Smart Films Enabled by Crosslinking Agent Optimization and MoS_2 Nanosheets for Energy-Saving Windows

Tao Yu[1], Fuman Jing[2], Yingjie Shi[2], Zhou Yang[1], Jianjun Xu[1], Zuowei Zhang[2*], Meina Yu[2*], Huai Yang[3*]

[1]School of Materials Science and Engineering, University of Science and Technology Beijing, Beijing, People's Republic of China

[2]Institute for Advanced Materials and Technology, University of Science and Technology Beijing, Beijing 100083, People's Republic of China

[3]School of Materials Science and Engineering, Peking University, Beijing 100083, People's Republic of China.

*Correspondence: Zuowei Zhang, zhangzuowei@bhu.edu.cn; Meina Yu, yumeina@ustb.edu.cn, Huai Yang, yanghuai@pku.edu.cn

1. Materials

The polyethylene glycol 200 diacrylate (PEGDA200), polyethylene glycol 400 diacrylate (PEGD400), polyethylene glycol 600 diacrylate (PEGDA600), lauryl methacrylate (LMA) and 2-Hydroxyethyl methacrylate (HPMA) were purchased from Shanghai Aladdin Biochemical Technology Co., Ltd. The Butyl Acrylate (BA) and hexyl acrylate (HA) were supplied by Shanghai Meryer Chemical Technology Co., Ltd. The commercial liquid crystal mixture E8 (ordinary refractive index $n_o = 1.527$, extraordinary refractive index $n_e = 1.774$, clearing point $T_{NI} = 345.2K$) was supplied by Yan Tai xian hua Chemical Technology Co., Ltd. The Irgacure 651 was supplied by Shanghai Aladdin Chemical Technology Co., Ltd. Ammonium molybdate ($(NH_4)_2MoO_4$, 99%), Thiourea (CH_4N_2S, 99%), glycerol ($C_3H_8O_3$, 99%), vitamin C (99%) and KOH (99%) were purchased from Sinopharm Chemical Reagent Co. DI water was obtained by purifying tap water from the lab. All chemical reagents have not been further purified and the chemical structure is shown in **Figure 1a**.

2. Characterization

SEM morphology: In order to analyze the structure of the polymer matrix, specimens were fragmented into small segments and immersed in cyclohexane for a duration of 20 days. Given the insolubility of the polymer matrix in the solvent, the liquid crystal (LC) content could be extracted by cyclohexane. Subsequently, the solvent was eliminated by subjecting the samples to heat in an oven at 75 °C for a period of 18 hours. Ultimately, the resultant polymer film was scrutinized through scanning electron microscopy (SEM, Hitachi S-4800, Hitachi Science System Ltd., Japan) subsequent to the application of a thin layer of gold onto the surface.

The microstructure of the materials was characterized by scanning electron microscopy, and the elemental distribution of the samples was using an X-ray energy dispersive spectrometer (EDS).

UV-VIS-NIR spectrum: A UV-VIS-NIR spectrophotometer (PerkinElmer Lambda 950) was used to characterize the transmission spectra of the films. The integral luminous transmittance (T_{lum}, 380 nm-780 nm) and solar transmittance (T_{sol}, 300 nm-2500 nm) were calculated from the transmittance spectrum of the film according to the following equations:

$$Tlum = \int \varphi lum(\lambda)T(\lambda)d\lambda / \int \varphi lum(\lambda)d(\lambda) \quad \text{(S1)}$$

$$Tsol = \int \varphi sol(\lambda)T(\lambda)d\lambda / \int \varphi sol(\lambda)d(\lambda) \quad \text{(S2)}$$

where T(λ) stands for the spectral transmittance, φlum(λ) is the standard luminous efficiency function of photopic vision within the wavelength range 380-780 nm, and φsol(λ) is the solar irradiance spectrum distribution for an air mass 1.5 corresponding to the sun standing 37°C above the horizon with 1.5 atm thickness at a solar zenith angle of 48.2°. The visible light transmittance variation (ΔT_{lum}) and the solar light modulation ability (ΔT_{sol}) are obtained by the following equation:

$$\Delta Tlum = Tlum(on) - Tlum(off) \qquad \text{(S3)}$$

$$\Delta Tsol = Tsol(on) - Tlsol(off) \qquad \text{(S4)}$$

Electro-optical measurement: An LC device parameter tester (LCT-5066C, Chang Chun Liangcheng Instrument Co. Ltd.) was used to examined the electro-optical characteristics at room temperature using 560 nm light. A square-wave modulated electric field (1000 Hz) was applied. The air transmission was normalized to 100%, whereas the dark transmittance was normalized to 0 %. The distance between the sample and the photodiode was 300 mm. The transmitted light intensity was measured at an angle of around ± 1 degree. The electro-optic performance characteristics of the PDLC film are discussed in depth. The threshold voltage (V_{th}) and saturation voltage (V_{sat}) contribute to 10% and 90% of the maximum transmittance, respectively. The transmittance of the film in the off-state and on-state are denoted by T_{off} and T_{on}, respectively. Contrast Ratio (CR) refers to the ratio of T_{on} to T_{off}. The rise time (T_R) is the time for the transmittance to reach 90% of the on-state transmittance, and the decay time (T_D) is defined as the time for the transmittance to reach 10% of the on-state transmittance.

XRD analysis: The structure of the MoS_2 nanosheets was characterized using X-ray diffraction (XRD, Rigaku-D/max 2550 PC, Japan). The chemical composition of the MoS2 structure was investigated using X-ray photoelectron spectroscopy (XPS, ESCA-LAB250).

FT-IR analysis: The MoS_2, KH550/MoS_2, and KH550 were fully ground with potassium bromide, and the samples were analyzed after tableting. The wavenumbers ranged from 4000 cm^{-1} to 400 cm^{-1} with a resolution of 1 cm^{-1}. FT-IR (Spectrum 3, PerkinElmer, Inc. America) analysis was used to characterize the silanization of MoS_2 surfaces. The emissivity spectra in the wavelength range of 2.5 to 14 μm were studied by Fourier transform infrared spectroscopy (FTIR) using a Nicolet iS50 instrument, USA.

3. Mechanical properties testing

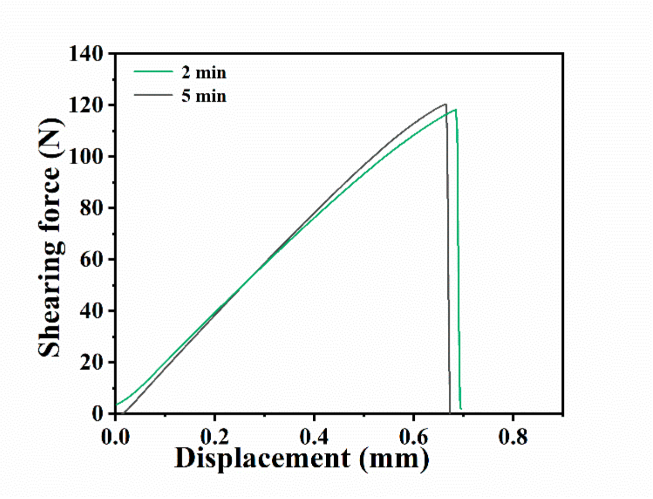

Figure S1. Mechanical properties of samples at different polymerization times.

4. EDS mapping of film.

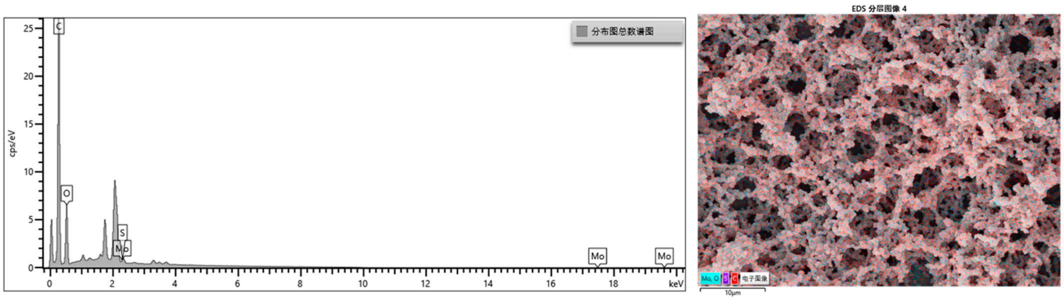

Figure S2. EDS layered images and element distribution maps.

Table S1. Distribution map total spectrum.

element	Wt%	Wt% Sigma
C	70.31	0.25
O	24.96	0.21
S	3.53	0.18
Mo	1.20	0.63
Total:	100.00	

5. Transmittance changes with viewing angle

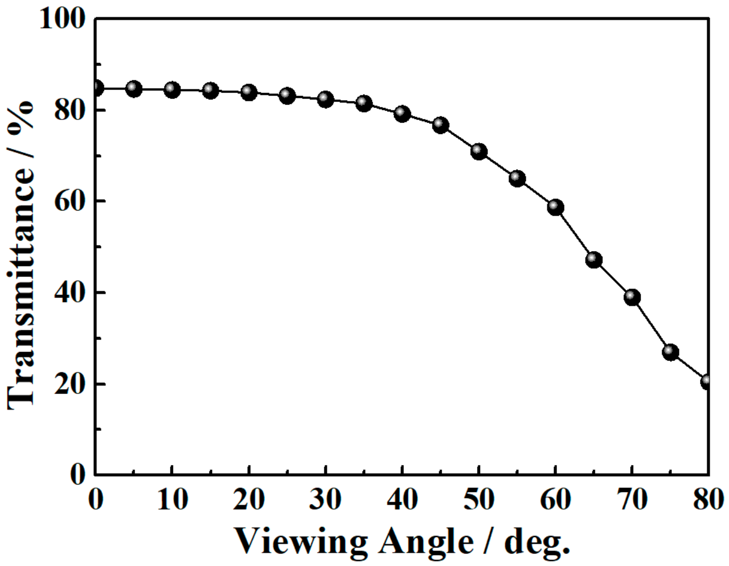

Figure S3. Transmittance changes with viewing angle.
